# Addressing Mental Health and Trauma-Related Needs of Sheltered Children and Families with Trauma-Focused Cognitive-Behavioral Therapy (TF-CBT)

**DOI:** 10.1007/s10488-022-01207-0

**Published:** 2022-07-22

**Authors:** Jamie A. Spiegel, Paulo A. Graziano, Emily Arcia, Shana K. Cox, Muriel Ayala, Nicole A. Carnero, Noelle L. O’Mara, Sundari Foundation

**Affiliations:** 1 Florida International University - Center for Children and Families and Department of Psychology, 11200 SW 8th Street, Miami, FL 33199 USA; 2 Emily Arcia Consulting Co., Miami, USA; 3 Sundari Foundation, Inc. dba Lotus House Women’s Shelter (Lotus House), 217 NW 15th Street, Miami, FL 33136 USA

**Keywords:** Homeless, Youths, Trauma, Trauma focused CBT, TF-CBT, Sheltered youth

## Abstract

Children and adolescents (“youth”) experiencing homelessness are at a disproportionately high risk of exposure to potentially traumatic events (PTE). However, limited evidence exists as to what interventions are effective when implemented with this high-risk population. The purpose of this study was to (1) document the mental health and trauma-related needs of sheltered youth and their mothers, and (2) examine the feasibility/effectiveness of Trauma-Focused Cognitive-Behavioral Therapy (TF-CBT) administered within the context of a homeless shelter. Three hundred and twenty-one youth (*M*_age_ = 10.06 years, *SD = *3.24 years, 56.4% male, 70.1% Black/African American, 34.6% Hispanic/Latinx) and their mothers were recruited from a homeless shelter and provided 10 weeks of TF-CBT, with the option for up to eight additional weeks of therapy based on clinical need. Families completed pre- and post-intervention assessments. Results demonstrated clinically elevated pre-intervention PTSD symptoms and rates of exposure to PTE in sheltered youth well above those previously reported in the general population. TF-CBT resulted in substantial reductions in both maternal and self-reported severity of youth PTSD symptomology, which were largely attributable to reductions in re-experiencing and arousal. Effectiveness of TF-CBT varied by age and the number of exposures to PTE. Overall, these findings illustrate the importance of assessing and addressing the mental health and trauma-related needs of sheltered youth and the feasibility and efficacy of embedding an evidence-based trauma-focused treatment protocol within a shelter environment. Additional implications of these findings are discussed.

Homelessness is a global problem impacting over 100 million people worldwide (United Nations Economic and Social Council, 2015). As of January 2020, the Department of Education^1^ reported that 1,508,265 children and adolescents (youth),^2^ enrolled in public schools, experienced homelessness (Vilsack et al., 2015). Retrospective data indicates that one in every 30 youth in the U.S., or 2.5 million youth (including both school age and below), experience homelessness annually (Bassuk et al., 2014). Although securing safe, adequate, and affordable access to basic needs, such as food and shelter are of primary importance, addressing the mental health needs of this marginalized and at-risk population is also essential. Studies completed over two decades ago found that up to 78% of youth experiencing homelessness have at least one mental health disorder (e.g., depression, behavior problems) and/or experience an academic or developmental delay (Committee on Community Health Services, 1996; Weinreb et al., 1998). However, less than one-third of these youth receive mental health treatment (Bassuk & Friedman, 2005). Given the disproportionately high rates of trauma experienced by youth experiencing homelessness (Cowal et al., 2002; Keeshin & Campbell, 2011; Masten et al., 1993), it is particularly important to investigate the feasibility and effectiveness of delivering evidence-based treatments for trauma and mental health issues within this population. Further, with so many youth entering our nation’s shelter systems (both domestic violence and homeless), shelter service providers that are appropriately resourced have an opportunity, through evidence-based assessment and therapeutic interventions, to provide targeted treatment for highly vulnerable, at risk youth. As such, they have a chance to transform what might otherwise be another layer of trauma into a window of opportunity for healing and growth.

## Trauma and Post Traumatic Responses in Youth Experiencing Homelessness

The DSM 5 defines a traumatic event as an instance in which an individual is directly or indirectly exposed to, witness to, or learns about a family member having been exposed to death or threat of death, actual or threat of serious injury, or actual or threat of sexual violence (American Psychiatric Association, 2013). In the general population, approximately 20% of youth are exposed to at least one potentially traumatic event (PTE) and approximately half of these youth experience polyvictimization (Alisic et al., 2014; McLaughlin et al., 2013; Saunders, 2003). The prevalence rates of traumatic events vary based upon the nature of the event. Ranging from 8 to 10% of youth experiencing at least one sexual assault (of which approximately half are reported to have occurred prior to age 13) to 38–70% of youth witnessing household or community violence prior to adulthood (Saunders & Adams, 2014).

Homeless youth are amongst the highest need children in our nation. Results of a recent meta-analysis indicate that 24% to 40% of school-age children experiencing homelessness had clinically significant symptoms of a mental health disorder, representing a rate of mental health disorders 2 to 4 times higher than those seen in low income homed peers (Bassuk et al., 2015). The prevalence rate of exposure to PTEs is substantially higher amongst youth experiencing homelessness than those found in the general population (e.g., Cowal et al., 2002; Keeshin & Campbell, 2011; Masten et al., 1993). Further, youth experiencing homelessness are up to three times more likely to experience PTSD than their homed peers (e.g., Stewart et al., 2004). However, studies to date have tended to focus on the prevalence of a single traumatic event exposure type making it difficult to determine the range of prevalence rates across exposure types in youth experiencing homelessness.

Reactions to PTEs are highly variable and depend upon the individual’s predispositions, the nature of the event, the duration and frequency of the event(s), and the reaction of the community (Caspi et al., 2002; Green et al., 1985; Toro et al., 1991). Post-traumatic reactions range from resiliency (Masten, 2011) to elevated symptoms or diagnosis of post-traumatic stress disorder (PTSD), separation anxiety, hyperactivity, inattention, and irritability (e.g., Bui et al., 2014). These reactions tend to be pervasive, impacting affect and mood (e.g., sadness, overly responsive to negative stimuli, lack of responsiveness to positive stimuli), behavior (e.g., avoidance, oppositionality, heightened fight or flight responses), cognition (e.g., self-blame, worthlessness, loss of trust; Cohen & Mannarino, 2008), ability to meet developmental milestones (e.g., Kaplan et al., 2016), and school readiness (Obradović et al., 2010). Even when full diagnostic criteria for PTSD are not met, subclinical PTSD symptomatology can result in substantial impairments (e.g., Carrion et al., 2002; Cuffe et al., 1998).

Variations in rates of PTSD diagnosis across studies (e.g., Alisic et al., 2014) can be explained by a variety of factors. Although lifetime incidence of PTSD tends to be greater for older as compared to younger youth (e.g., Finkelhor et al., 2007; Grasso et al., 2013), the rate at which youth develop PTSD following exposure to a traumatic event are fairly consistent across development^3^ (Fletcher, 1996). However, variations in rates of PTSD can be seen as a product of the type of traumatic event examined (Alisic et al., 2014) and the way in which PTSD is assessed. For instance, approximately 6% of children exposed to natural disasters develop PTSD (McLaughlin et al., 2013; Shannon et al., 1994) whereas as many as 90% of youth exposed to sexual abuse develop PTSD (Nurcombe, 2000; Walker et al., 2004). Further, greater number of exposures to traumatic events (polyvictimization) are associated with greater severity of PTSD symptoms and impairment (Finkelhor et al., 2007, 2009). Regarding assessment type, several studies have demonstrated poor inter-rater consistency regarding types of exposures to PTEs and presence of PTSD symptomology (Ceballo et al., 2001; Oransky et al., 2013; Stover et al., 2010). Meta-analytic findings indicate a rate of diagnosis of 17% for youth self-report as compared to a rate of 5% for parental report (Alisic et al., 2014).^4^ These findings highlight the importance of considering frequency and nature of trauma exposure and informant type in the examination the diagnosis and treatment of post traumatic reactions.

## Heightened Vulnerability of Homeless Youth

Although the DSM 5 does not recognize homelessness in and of itself as a criterion A stressor for PTSD (American Psychiatric Association, 2013), substantial research has demonstrated that homelessness is a complex life stressor which increases the risk of developing mental health difficulties (Goodman et al., 1991). The majority of youth respond to the stress of homelessness with, at a minimum, worries about the safety of themselves and their families (National Center on Family Homelessness, 1999). Homelessness often results in a substantial disruption to daily routines and the removal of social supports as one leaves familiar surroundings, possessions, familial support systems, and communities. Additionally, transitioning to living in a shelter often results in a decreased sense of privacy, safety, predictability, and control (Kirkpatrick & Byrne, 2009).

In addition to increasing the stress of daily living, homelessness increases exposure to Adverse Childhood Events (ACES; Felitti et al., 1998). The ACES model of trauma posits that childhood exposure to adverse events has a direct negative impact on long-term mental and physical health (Cronholm et al., 2015; Felitti et al., 1998). Substantial evidence suggests that, although at least half of the population is exposed to one or more ACES (Felitti et al., 1998), youth exposed to four or more ACES are up to five times more likely to develop mental health disorders (e.g., Hunt et al., 2017; Merrick et al., 2017). Approximately 12.5%, or one in every eight youth, experience high levels of ACES (≥ 4; Felitti et al., 1998).

Youth living in poverty are at substantially greater risk of experiencing high levels of ACES (Halfon et al., 2017). In fact, living below the poverty line results in a four hundred percent increase in the risk of exposure to high levels of ACES (≥ 4), as compared to individuals from financially stable homes (Halfon et al., 2017). However, in addition to exposure to poverty, youth experiencing homelessness also report a history of greater exposures to separation from caregivers and exposure to the foster care system (Zlotnick, 2009; Zlotnick et al., 1998), community violence, decreased access to health care and educational services (Masten et al., 1997; Panter-Brick, 2004; Shelton et al., 2015; Zlotnick, 2009), exposure to parental substance abuse (e.g., Stein et al., 2002), and parental incarceration (Casey et al., 2015; Wildeman, 2014). This increased exposure to ACES culminates in an elevated risk for the development of emotional disorders, amongst other medical and mental health difficulties, for those youth experiencing homelessness. Indeed, the National Child Traumatic Stress Network reports that “more than one-fifth of homeless preschoolers have emotional problems serious enough to require professional care” (Bassuk & Friedman, 2005, p. 2) and approximately 18% of youth experiencing homelessness meet criteria for PTSD (Stewart et al., 2004).

## Trauma Informed Care in Youth

Although youth experiencing homelessness represent a particularly vulnerable population for whom early assessment is critical and intervention addressing mental health and trauma are vital, limited research has examined the efficacy of interventions addressing the needs of this population. Left untreated, youth responses to trauma exposure can follow a chronic course (Bolton et al., 2000; Morgan et al., 2003; Yule et al., 2000). Although several therapeutic approaches have been proposed for the treatment of post traumatic symptoms in youth, Trauma-Focused Cognitive-Behavioral Therapy (TF-CBT; Cohen et al., 2016) has consistently emerged as a gold standard. Specifically, meta-analytic findings indicate that TF-CBT outperforms all other commonly utilized forms of therapy (e.g., EMDR, supportive counseling family therapy, parent training) in the treatment of post-traumatic responses in youth (Mavranezouli et al., 2020). Although originally designed to treat post-traumatic stress, TF-CBT has also demonstrated efficacy in the reduction of symptoms of depression, anxiety, and trauma-associated behavior difficulties in 3–17-year-old youth (Cary & McMillen, 2012; Gutermann et al., 2016; Kowalik et al., 2011; Morina et al., 2016; Silverman et al., 2008). Specifically, although the greatest effects of TF-CBT can be seen in the reduction of PTSD symptoms, meta-analytic findings indicate that externalizing behaviors are also substantially reduced by TF-CBT (*d* = 0.67; Hoogsteder et al., 2021). These findings are particularly important given evidence that (a) youth who experience trauma are at an increased risk for engaging in externalizing and delinquent behaviors (e.g., Becker & Kerig, 2011; Fitton et al., 2020), (b) youth with externalizing behavior disorders are at an increased risk for experiencing PTEs (e.g., Maschi et al., 2013), and (c) in youth PTSD symptoms commonly present as oppositionality (e.g., American Psychiatric Association, 2013).

TF-CBT is largely based upon a gradual exposure model. Youth and their parents are taught coping strategies and then face exposures of gradually increasing intensity as they move through a phase-based manualized treatment. Sessions are conducted in separate child- and parent-sessions as well as conjoined child-parent sessions (Cohen et al., 2010). Because youth’s abilities to talk about and cope with challenging experiences are often dependent upon the adults in their lives (Fivush, 1998; Mash & Terdal, 1997) and because their emotional expression and coping strategies are largely dependent upon the behavior of adults in their lives (Eisenberg et al., 1996), each component of TF-CBT is provided to both the youth and their parent in parallel sessions.

To date, the efficacy of TF-CBT has been demonstrated in samples of youth exposed to sexual abuse (Cohen & Mannarino, 1996; Cohen et al., 2004a, b, 2005; King et al., 2000), domestic violence (Cohen, 2005), violence outside the home (Stein et al., 2003), death of a loved one (Cohen et al., 2004b, 2006), terrorist attacks and wars (Ertl et al., 2011; Hoagwood et al., 2006; Ruf et al., 2010), natural disasters (Berger & Gelkopf, 2009; Pityaratstian et al., 2015), motor vehicle accidents (Meiser-Stedman et al., 2008; Smith et al., 2007) and polivictimization (Auslander et al., 2017). However, to date, research on the effectiveness of TF-CBT in the treatment of youth experiencing homelessness is fairly limited. Wenocur et al. (2016) reported on the practicality of implementing TF-CBT in an emergency family housing setting, however the sample of children who completed treatment within a three-year period was small (*N* = 29). Nonetheless, they reported that 86% of youth self-reported reduced trauma-related symptomatology and 97% of mothers reported reduced problem behaviors (Wenocur et al., 2016). Although these findings offer preliminary support for utilizing TF-CBT with sheltered populations, the Wenocur study reports on a small sample size and largely relies on qualitative reports of treatment efficacy. To our knowledge, no other study has examined the effectiveness of TF-CBT in reducing trauma-related symptoms in sheltered youth.

## Present Study

The life circumstances and events occurring prior and leading to homelessness, the overall elevated risk of trauma exposure in youth experiencing homelessness, the long-term effects of trauma, and the lack of research on how best to address trauma-responses in this population, culminate in an urgent need to better understand the mental health and trauma experiences of sheltered youth and to establish evidence-based intervention practices for this vulnerable population.

As part of a larger community-based service driven research project, the current study sought to (1) examine cross informant reports of mental health difficulties, prevalence of exposure to PTEs, and diagnostic status within a large sample of youth experiencing homelessness at entry into a women’s homeless shelter, (2) determine the feasibility and effectiveness of implementing TF-CBT within the context of a homeless shelter, and (3) examine youth-level constructs (i.e., age/grade and number of exposures to PTE types) which may moderate treatment effectiveness.

It was hypothesized that both the prevalence rates of exposure to PTEs and PTSD diagnoses would be higher for youth experiencing homelessness as compared to the prevalence rates found in the general population. Based upon previous studies demonstrating variability of parent and youth self-report of PTSD symptomology (e.g., Alisic et al., 2014), it was further hypothesized that youth self-report would indicate a higher prevalence of post-traumatic symptomology as compared to maternal report. It was also hypothesized that both trauma and externalizing symptoms in youth would be improved substantially from TF-CBT. Based upon evidence that youth across development respond similarly to exposure to trauma (Fletcher, 1996) and that TF-CBT has demonstrated efficacy across development (as long as the child has verbal capacity and memory of the trauma; e.g., Kliethermes et al., 2017; Pollio & Deblinger, 2017), it was hypothesized that TF-CBT would be equally effective for youth regardless of age. However, age/grade was examined as a moderator to replicate prior results within a sample of youth experiencing homelessness. Finally, given evidence that polyvictimization is associated with more severe PTSD symptomology and trauma-related impairment (Finkelhor et al., 2007, 2009), it was hypothesized that greater exposure to PTEs would be associated with poorer treatment responses.

## Methods

### Participants and Recruitment

The present study was part of a larger service driven, community-based, research project conducted at the Lotus House, the largest women’s shelter in the state of Florida and one of the largest in the nation, with a nightly capacity to shelter over 500 women and youth. Data were collected between June of 2017 and July of 2020. All families were offered clinical assessments and therapeutic services based on clinical need promptly upon admission. To qualify for the current study, families were required to (a) have a youth between the ages of 5 and 17-years-of-age who (b) spoke English, Spanish, and/or Creole. Although not all youths reported exposure to a PTE,^5^ all youth entering the shelter had experienced at least two substantial ACES (i.e., homelessness and poverty). As such, all youths were likely to benefit from trauma-informed intervention (i.e., trauma narrative could focus on PTE or ACEs) and no explicit inclusion/exclusion criteria regarding severity of trauma-related symptomology were implemented for this study. Families were permitted to receive clinical services without participating in research; however, almost all mothers whose youth received services provided consent to participate in research. Exclusionary criteria, for the current study, included youth (a) outside the target age range (younger than 5 years old^6^), (b) already receiving therapeutic services elsewhere, and (c) for whom clinical judgement dictated that presenting difficulties would be better addressed by Parent Child Interaction Therapy (Eyberg et al., 2001; i.e., prominent externalizing behavioral difficulties in children 5–7 years old only). As TF-CBT entails both youth and parent sessions, all mothers participated in their youth’s treatment.

The sample for the present study consisted of **321** youth between the ages of 5 and 17 (*M*_age_ = 10.06, *SD* = 3.24) whose mothers provided consent to participate in the study. The sample was comprised of mostly males (56.4%) and Black/African Americans (70.1%). Of these youth, 24 never attended treatment; leaving **297** youth who received at least one session of TF-CBT (intent to treat sample). Of these 297 participants, **214** completed treatment (i.e., 10 or more sessions/75% of treatment/completion of the trauma narrative).

Of the 297 participants in the intent to treat sample, the mean age was 10.03 years (*SD* = 3.21 years), and most were male (56.2%) and Black/African American (69.4%), and 34.0% were Hispanic/Latinx. See Table 1 for other descriptive sample data. Descriptive data for the 214 treatment completers can also be found in Table 1. Chi square difference tests indicated no demographic differences between those youth who completed treatment as compared those who dropped out of treatment prior to completion (i.e., prior to completing at least 75% of the intervention/completion of the trauma narrative).

**Table 1:** Participant baseline demographic variables by initial intervention assignment

	Intent to treat (*N* = 297)	Completers (*n* = 214)
*Demographic variables*		
Child sex (% female)	43.8	43.0
Child mean age	10.03 (3.21)	10.16 (3.23)
Youth education (%)		
Preschool	3.0	3.2
Elementary school (K-6)	72.4	70.6
Junior high-high school	23.6	25.2
GED	1.0	0.9
Child Race (%)		
Black	69.4	67.8
White	29.6	30.8
Biracial/other	0.7	1.4
Child Ethnicity (%)		
Latinx White	29.0	29.9
Non-Latinx White	2.0	2.8
Latinx Black	4.7	3.3
Non-Latinx Black	62.3	62.1
Maternal education (%)		
Some high school	32.3	32.2
High school diploma/GED	45.5	47.2
Technical degree	3.0	3.3
Some college	12.8	11.2
Associate’s degree	1.0	0.5
Bachelor’s degree or higher	5.4	5.6
Maternal employment status (%)		
Unemployed	72.7	73.8
Employed	27.3	26.2
Home Language (%)*		
Monolingual (English only)	72.4	73.4
Monolingual (Spanish only)	10.8	9.3
Monolingual (Creole only)	0.3	0.5
Bilingual (Spanish/English)	15.2	15.4
Bilingual (English/other language)	1.3	1.4
Department of child and families involvement (%)		
Present	13.1	13.6
Past	33.7	32.7

Values enclosed in parentheses represent standard deviations

*Home Language here references language used at home by the caregiver when speaking to the child. Child’s preferred language often varied from that spoken in their homes/families

The 297 youth included in this study were from 224 families, with 58 sets of siblings within the sample. The number of youths in the families ranged from one to seven, with an average of 2.7 youth in each family (although not all youth qualified for intervention). In the majority of families, the home language was English (72.4%); another 26% spoke Spanish exclusively or were bilingual. Clinical services were provided to youth and their mother/guardian in their preferred language which included English, Spanish, and/or Creole. Mother/guardian age ranged from 23 to 66 years (*M* = 35.09, *SD* = 8.31). The majority of mothers were unemployed at the onset of treatment and the modal family income was less than 10,000 annually, with only 2% of mothers reporting income greater than 25,000 annually.

### Study Design and Procedure

This study was approved by the University’s Institutional Review Board. Clinicians at the Lotus House who delivered the interventions, in the family’s preferred language, were master’s level clinical staff or therapists, licensed or registered for licensure, and certified or in the process of receiving their certification in TF-CBT. Counselors received weekly supervision by the shelter’s program clinical director, a state qualified psychologist and social worker trained and certified in TF-CBT.

Upon admission to the shelter, clinicians and trained staff administered an assessment protocol that lasted approximately 2 h and included: (a) a biopsychosocial interview of mothers that gathered relevant background information on the youth and family, (b) questionnaires on youth’s externalizing behavior problems, trauma histories, and symptoms, (c) questionnaires on maternal stress, and (d) videotaped observations of three 5-min standard parent–child interaction situations that varied in the degree of parental control expected (for youth ages 6 months to 12 years 11 months). See below for a description and names of assessments. Families completed a similar post-intervention assessment upon completion of intervention (i.e., 10 sessions) or 4 months after the start of the intervention (mean time to complete intervention = 3.67 months, *SD* = 1.25 months). Families were given small incentives such as a small toy to the youth, or a small gift to the parent upon completion of the assessments, and all interventions were provided at no cost. Staff conducting the pre- and post-intervention assessments were not the same clinicians who provided therapeutic services.

### Intervention Description and Adaptation

TF-CBT (Cohen et al., 2017; Kliethermes et al., 2017) is an evidence-based program designed for the treatment of trauma-related symptoms in youth ages three to eighteen years. In TF-CBT youth and their parents are taught coping strategies and face exposures of gradually increasing intensity as they move through treatment. Therapy sessions follow a preset sequence (PRACTICE): psychoeducation and parenting skills, relaxation skills, affective regulation skills, cognitive coping skills, trauma narrative and cognitive processing of the traumatic events, in vivo mastery of trauma reminders, conjoint youth-parent sessions, and enhancing safety and future developmental trajectory (Brown et al., 2020; Cohen et al., 2010). Treatment is broken down into three phases (a) stabilization, (b) integration, and (c) consolidation. TF-CBT is traditionally completed within 12–15 sessions with approximately equal sessions for each of the three phases. In cases of more complex trauma, treatment can be extended to up to 16–25 sessions (Cohen & Mannarino, 2016).

For this study, given the transient nature of the sheltered population (Culhane et al., 2007), every effort was made to complete TF-CBT within 10–12 sessions. There was some variability in the number of sessions spent within each phase of treatment, largely based upon clinical need and youth uptake and integration of skills. However, in general 4–5 sessions were spent within phase one (i.e., Safety and Stabilization), 4–5 sessions within phase two (i.e., Formal Gradual Exposure), and 1–3 sessions on phase three (i.e., Consolidation and Integration) of TF-CBT. During the tenth session or 4 months after initiating treatment (whichever was earlier), youth, their mothers, and their therapist met to determine whether treatment goals were met or if additional sessions were needed. If therapeutically necessary, youth received additional sessions (*n* = 46), not exceeding 18 sessions.

### Measures of Feasibility and Acceptability

#### Intervention Completion and Attendance

Attendance for each session was measured from therapists’ notes within the shelter’s electronic medical records. Trauma narratives were completed at or before the ninth intervention session. Given the importance of completion of the trauma narrative for reduction of PTSD symptomology (Deblinger et al., 2011), intervention completion rates were calculated based on the completion of at least 10 sessions.

#### Consumer/Intervention Satisfaction

Parents provided ratings of satisfaction at post-intervention by completing selected items from the Therapy Attitude Inventory (Brestan et al., 1999). Raters indicated on a five-point Likert scale their degree of satisfaction of (a) improvements in the parent-youth relationship (b) progress the youth made in general behavior, (c) progress the youth made in trauma symptoms, (d) general feeling about the program, and (e) how likely they were to recommend the program to others. The mean level of satisfaction was calculated across these five items (α = 0.75 for maternal report).

### Youth Outcomes

#### Grade

Grade was dichotomized into (a) kindergarten through sixth grade and (b) seventh through twelfth grade to allow for comparisons between youth in elementary and junior high/high school.

#### Child and Adolescent Trauma Screen (CATS)

Mothers completed the CATS-caregiver for all youth and youth ages eight and older completed the CATS-youth at pre- and post-intervention. The CATS assesses for exposure to 14 PTEs (and allows for short response of any additional potential traumas) as well as the frequency of each of the 20 post-traumatic symptoms (only 16 symptoms were assessed for children under 7 years old), based upon the DSM-5 criteria (American Psychiatric Association, 2013). *Rate of exposure to PTE types* was calculated by summing the total number of PTE types endorsed by the youth or their mother. As such scores ranged from 0 to 14. It is important to note that although homelessness and poverty are ACEs/childhood risk factors, these were not included within the total number of PTE types because (a) neither homelessness nor poverty meet DSM 5 criteria for a PTE and (b) all participants in the sample experienced homelessness and poverty and as such including these variables would not result in any variance within the sample.

##### Severity, Symptomology, and Diagnostic Status

Post-traumatic symptoms were rated on a 4-point Likert-scale ranging from 0 (never) to 3 (almost always), resulting in a single total severity score, with higher scores indicating greater severity of post-traumatic stress (Parent CAT α = 0.83; Self CAT α = 0.86). Endorsement rates of (a) reexperiencing, (b) avoidance, (c) negative mood and cognitions, and d) arousal symptoms (which coincide with criterion B-E of the DSM 5 PTSD symptom criterion, respectively) were calculated by summing the number of items within each domain rated as occurring half the time (2) or almost always (3). Higher scores indicate greater presence of symptomology. Finally, diagnostic status was calculated by determining whether endorsement rates of reexperiencing, avoidance, negative mood/cognition, and arousal met or exceeded DSM criteria (i.e., presence of at least 1, 1, 2, 2, and 1 symptom, respectively) for those youth with reported exposure to at least one PTE. Diagnostic status was coded dichotomously based upon meeting or not meeting DSM 5 criteria for PTSD.

#### Eyberg Child Behavior Inventory (ECBI)

Mothers completed the ECBI (Eyberg & Ross, 1978), a 36-item questionnaire designed to assess externalizing behavior problems in youth ages 2–16 years, at pre- and post-intervention. The raw score from the total intensity scale was used in the present study as a measure of externalizing behavior problems (α = 0.94).

### Data Analytic Plan

All analyses were conducted using Statistical Package for the Social Sciences, version 20 (SPSS 26). At pre-intervention 6.76% of data were missing. At post-intervention 39.71% of data were missing, primarily due to families exiting the shelter prior to completion. Based upon Little’s Missing Completely at Random test, data at pre-intervention χ^2^(14) = 38.06, *p* < 0.001 and post-intervention χ^2^(8) = 19.19, *p* < 0.05 were not missing at random. Therefore, as recommended in clinical trials, intent to treat analyses with the use of multiple imputation was used (Little & Yau, 1996; Rubin, 1988; Von Hippel, 2020) in addition to analyses for youth who completed treatment.

In an effort to provide updated incidence/prevalence rates within a sample of youth currently experiencing homelessness, initial analyses focused on describing the percentage of youth who were clinically elevated in terms of behavior problems and trauma symptoms as well as the percentage of youth who experienced PTEs at pre-intervention. Paired-sample t-tests were utilized to examine whether prevalence rates varied by reporter (i.e., self- versus maternal-repot). Next, completion, attendance, and intervention satisfaction were examined. For the primary analyses, multiple repeated measures ANCOVAS controlling for age were conducted to examine pre- post-intervention changes for youth outcomes, including changes in diagnostic status. Other demographic variables were not included as covariates, as age was the only variable consistently significantly correlated to the outcomes of interest.^7^ A series of repeated measures ANOVAS were conducted to examine the potential moderating effect of grade level on pre- to post-intervention outcomes. Finally, a series of repeated measures ANCOVAS controlling for age were conducted to examine the moderating effect of the number of exposures to PTE types (based on maternal report on the CAT), on pre- to post-intervention outcomes.

## Results

### Cross Informant Reports of Mental Health Difficulties at Pre-intervention

With regard to externalizing behavior symptoms, 24.80% of youth had a total score in the clinical range on the ECBI (i.e., score of 131 or higher), based upon maternal report. In terms of trauma symptoms, 52.9% of the youth had total raw scores in the clinically elevated range (i.e., score of 12 or higher for ages 5–6 years and 15 or higher for ages 7 and older on PTSD severity) as reported by mothers on the CATS. Based on self-report, 69.52% of youth had scores in the clinically elevated range on the CATS.

Rate of exposure to PTEs are outlined in Table 2. Overall, the results indicate that regardless of reporter, the most commonly experienced traumatic events were witnessing violence (at home or in the community) and sudden or violent death of a loved one. Self-report consistently indicated a higher rate of exposure to PTEs than did maternal report, with the exception of witnessing someone in the family get slapped, punched or beat up, which mothers endorsed more often than did youth. Paired-sample t-tests were conducted to determine whether discrepancies between maternal and self-reports were significantly different. Given the number of analyses conducted Holm’s Step-Down Procedure (Holm, 1979) was implemented to minimize Type 1 error. Results, depicted in Table 2, indicate that mothers reported significantly greater incidence of witnessing violence in the home (t = 3.28, *p* < 0.001) than did youth, but youth reported significantly greater witnessing of community violence than did their mothers (t =  − 10.15, *p* < 0.001). Youth also reported greater incidence of physical abuse, emotional abuse, separation from a caregiver (including abandonment by father), and serious accident or injury as compared to maternal report (*p*s < 0.001).

**Table 2:** Number of youths exposed to potentially traumatic events based on maternal and self-report at pre-intervention

Trauma type	Parent CAT All ages (N = 297)	Parent CAT 8-17 years (N = 214)	Self CAT 8-17 years (N = 214)	t-test
Burglary/robbed	2	1	5	− 1.64
Natural disaster	68	49	63	− 2.43*
Serious accident/injury	68	50	77	− 4.16***
Death of loved one	163	126	123	0.60
Scary medical	42	31	29	0.50
War	1	1	1	–
Any physical abuse	57	37	78	− 3.67***
Physical abuse—family	29	19	32	− 3.04**
Physical abuse—nonfamily	21	13	46	− 5.10***
Attacked	8	6	15	− 2.53*
Any witnessed violence	172	125	163	− 4.89***
Witness physical violence family	132	93	71	3.28***
Witness community violence	61	49	137	− 10.15***
Witness others attacked	52	38	49	− 1.99*
Any sexual abuse	22	21	24	− 0.83
Forced sexual touching	20	19	22	− 0.90
Forced sexual pressure	6	5	7	− 0.82
Other traumatic event				
Emotional abuse	35	24	20	− 7.08***
Separation from parent	66	44`	65	− 3.28***
Bullying	104	72	86	− 2.04*
PTSD Severity Score Mean (SD)	17.15 (10.66)	17.18 (10.86)	21.76 (11.77)	− 7.08***

T-tests compare maternal- and self-reports on the CAT for participants 8–17 years old. Bold = significant after Holm’s stepdown correction procedure

*Any Physical Abuse* experienced at least one instance of either physical abuse from a family member or nonfamily member or has been attacked, *Any Witnessed Violence* witnessed at least one instance of either physical abuse of a family member or nonfamily member or has witnessed an attack, *Any Sexual Abuse* experienced at least one instance of either forced sexual touching or sexual pressure. *Other Traumatic Event* events endorsed as part of an open-ended question of other traumatic events not traditionally listed by the CATS

*p < 0.05, **p < 0.01, ***p < 0.001

Although the possible range of scores for rate of exposure to PTE types (rate of polyvictimization) was 0–14, in this sample no youth endorsed experiencing exposures to more than ten exposure types. Based upon maternal report, the mean number of traumatic event type exposures was 2.26 and the majority of mothers endorsed between one and three exposure types, with 21.9% of mothers reporting exposure to ≥ 4 PTE types. Self-report indicated that the mean number of exposure traumatic event types was 3.14, with the majority of youth endorsing two to four exposure types. Approximately 38% of youth endorsed exposure to ≥ 4 PTE types.

### Feasibility and Acceptability of TF-CBT

**Fig. 1:**
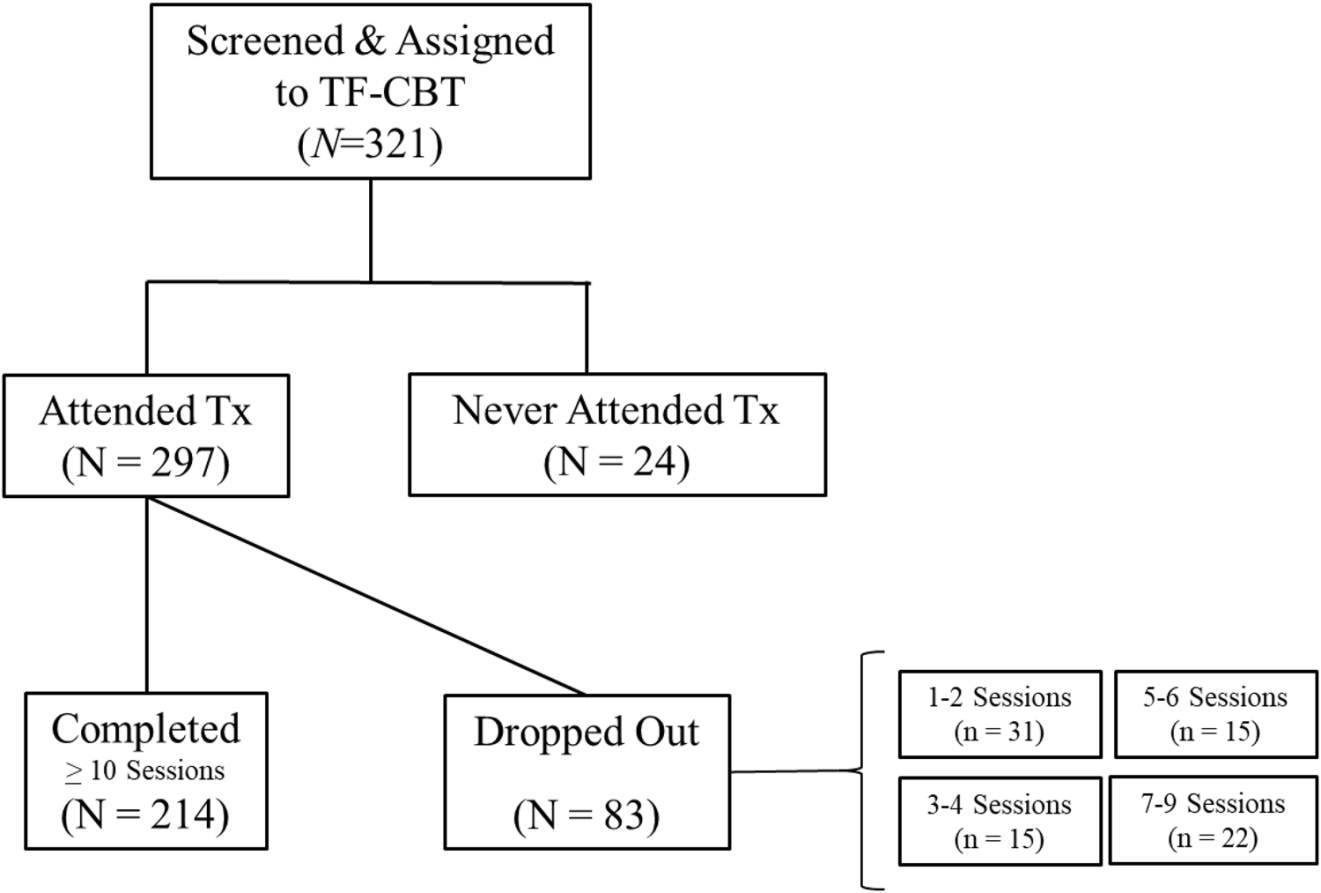
Consort flow diagram for youth assigned to TF-CBT based upon clinical need. *TF-CBT* trauma-focused cognitive-behavioral therapy, *Tx* treatment

To determine the feasibility and acceptability of implementing TF-CBT within the context of a homeless shelter, rates of treatment completion, treatment fidelity, and satisfaction with treatment were examined. In terms of intervention completion, 7.48% of families never initiated TF-CBT after assessment and assignment (see Fig. 1). Of the families that initiated intervention, 72.05% of families in (*n* = 214) completed all intervention sessions (i.e., 10 or more sessions). The primary reason for treatment dropout (96.7%) was leaving the homeless shelter. Maternal report (*M* = 4.27, *SD* = 0.69) indicated high levels of satisfaction with treatment.

A random 40% of sessions (10 sessions each for 60 participants who completed TF-CBT) were selected and scored on treatment fidelity. Specifically, treatment notes were reviewed to determine adherence to treatment protocol (i.e., covering PRACTICE sequence) and to ensure parents were seen by the therapist in addition to youth. Results indicated that in all but one session for one participant therapists met with parents to instruct them on topics covered within session, in addition to meeting individually with the youth. With regard to adherence to the treatment protocol, the PRACTICE sequence was adhered to in 96% of sessions reviewed.

### Efficacy of TF-CBT

As indicated in Table 3, TF-CBT resulted in significant reductions in the severity of PTSD-related symptoms, such that there was a significant reduction in youth who fell within the clinically elevated range on the CAT from pre- to post-intervention based on both maternal (F = 19.41, *p* < 0.01) and self-report (F = 7.58, *p* < 0.01). Based on maternal and self-report, TF-CBT was most effective at reducing criterion B (reexperiencing) symptoms (*p* < 0.01). Maternal report also indicated a significant reduction in criterion E (arousal) symptoms (*p* < 0.001). TF-CBT also resulted in significant reductions in maternal report of externalizing behaviors as measured by the ECBI intensity scale (*p* < 0.001).

**Table 3:** Repeated measures ANCOVAs examining pre-post intervention scores covarying for age

	Intent to treat F	Completers F
Parent CATS		
Symptom Severity	12.10***	12.91***
Reexperiencing Sx	7.84**	3.88*
Avoidance Sx	5.32*	6.98**
Neg Mood/Cog Sx	0.41	0.75
Arousal Sx	12.87***	10.55**
Self-report CATS		
Symptom Severity	7.44**	6.09*
Reexperiencing Sx	7.92**	6.32*
Avoidance Sx	0.29	0.01
Neg Mood/Cog Sx	0.34	0.01
Arousal Sx	0.05	0.01
ECBI		
Intensity Raw	29.48***	22.65***
CAT Scores in PTSD Range		
Maternal report	19.41**	17.87***
Self-report	7.58**	7.00**

Effects in bold remained significant after Holm’s stepdown procedure

*Sx* symptom count

*p < 0.05, **p < 0.01, ***p < 0.001

### Moderation of Treatment Effectiveness

As indicated in Table 4, grade level (i.e., K-6th versus 7–12th grade) significantly moderated the effects of TF-CBT on externalizing behavior (*p* < 0.001), but not trauma-related symptoms. Specifically, children in elementary school demonstrated greater pre-post changes in ECBI scores than did junior high and high school students. In contrast, the total number of exposures to PTE types, moderated the effects of TF-CBT on maternal and self-reported severity of trauma symptoms (*ps* < 0.001; Table 5), but not externalizing behaviors. Specifically, sheltered youth who did not report exposure to any PTE types did not show a significant reduction of trauma symptoms. However, both youth experiencing less than four and four or more exposures to PTE types demonstrated improvements pre- to post- intervention, with youth experiencing four or more exposure types consistently benefiting more from TF-CBT than youth with exposure to less than 4 exposure types. Notably, post-intervention trauma severity scores were equivalent across groups.

**Table 4:** Results of repeated measures ANOVA analyses examining the effects of grade on parent and child outcomes of TF-CBT in the intent to treat sample

	Elementary *M* (SD) *n* = 232		Junior high/ high school *M* (SD) *n* = 80		Time Effect F	Time × Group Effect F
	Pre	Post	Pre	Post		
Parent CATS Sx Severity	17.06 (12.10)	11.34 (9.96)	18.25 (19.79)	14.29 (16.29)	20.50***	0.67
Self-report CATS Sx Severity	22.10 (14.73)	13.40 (8.83)	21.45 (19.49)	13.45 (11.69)	53.02***	0.10
ECBI Intensity Raw	107.50 (49.52)	86.81 (43.78)	83.84 (85.20)	100.43 (75.34)	0.19	15.63***

*Sx* symptom

***p < 0.001

**Table 5:** Results of repeated measures ANCOVA analyses examining the effects of number of exposures to potentially traumatic events on parent and child outcomes of TF-CBT controlling for age in the intent to treat sample

	0 PTE *M* (SD) *n* = 37		1–3 PTE *M* (SD) *n* = 214		> 4 PTE *M* (SD) *n* = 70		Time Effect F	Time × Group Effect F
	Pre	Post	Pre	Post	Pre	Post		
Parent CATS Sx Severity	12.27 (32.35)	10.67 (27.05)	16.48 (12.50)	12.59 (10.44)	21.40 (21.91)	11.63 (18.32)	23.19***	5.55**
Self-report CATS Sx Severity	15.41 (40.38)	14.85 (28.33)	20.81 (13.80)	13.89 (9.59)	26.79 (24.26)	13.53 (17.00)	24.10***	5.86**
ECBI Intensity	96.01 (123.30)	93.84 (105.69)	98.04 (50.40)	88.64 (43.06)	104.14 (87.75)	92.27 (75.19)	8.28**	0.45

*PTE* potentially traumatic event exposure, *Sx* symptom

*p < 0.05, **p < 0.01, ***p < 0.001

## Discussion

The current study provides updated estimates of the clinical needs and prevalence of exposure to PTEs in sheltered youth. To our knowledge, the present study represents the first large scale quantitative investigation of the mental health needs and trauma histories and symptoms of sheltered homeless youth and the feasibility and efficacy of providing Trauma-Focused-CBT to address those needs. The findings demonstrate both the magnitude of the needs of sheltered youth, the feasibility of providing evidence-based interventions within the context of a homeless shelter, and the value of providing such services to sheltered youth and their families. Further, the time-limited adaptation of TF-CBT, a necessary adaptation given the transient nature of the homeless population (Culhane et al., 2007), was found to be efficacious at reducing trauma-related symptomology. This offers a promising blueprint for other shelters and community mental health providers to follow in their provision of clinical services in the future. Finally, although TF-CBT resulted in a significant reduction of symptomology across youth, it was more effective at reducing externalizing behaviors in children as compared to adolescents and was more effective at reducing symptomology in youth who had experienced greater PTE exposure types. These findings are discussed in greater detail below.

### Profiles of Symptomatology and Trauma Exposure at Pre-intervention

Whereas previous studies have indicated that the prevalence rate of exposure to PTEs is substantially higher for youth experiencing homelessness (e.g., Cowal et al., 2002; Keeshin & Campbell, 2011), findings from this study suggest that a more nuanced examination of prevalence rates is needed. Specifically, as compared to prevalence rates reported by Saunders and Adams (2014), in the present study youth experiencing homelessness reported experiencing similar rates of exposure to at least one instance of physical abuse, sexual abuse, natural disasters, and serious accidents (e.g., car accidents) prior to being sheltered. However, the prevalence of witnessing physical violence in the home or community and exposure to bullying were reported at greater rates in this study than in studies with homed youth. The fact that the study was conducted at a women’s homeless shelter, rather than a gender-neutral shelter, may have impacted the reported rates of witnessing violence in the home. Specifically, violence is a leading cause of homelessness for women–with fifty-seven percent (57%) of all women experiencing homelessness reporting domestic violence as the immediate cause of their homelessness (National Alliance to End Homelessness, 2019). The prevalence of violence for women at the women’s shelter was nearly ubiquitous with ninety-nine percent (99%) of the incoming women and children at the Lotus House reporting victimization from domestic and/or intimate partner violence, gender-based violence, trafficking and other crimes and serious trauma. However, it is important to note that this study did not include a homed comparison group and as such, comparisons rely on prevalence rates reported in previous studies.

Consistent with previous studies (Saunders, 2003), maternal report in this study indicated that approximately two thirds (63.8%) of youth experiencing homelessness experienced two or more PTE types. These rates were substantially higher when self-report was examined. Specifically, 85.6% of youth reported exposure to two or more PTEs. Importantly, these rates may underestimate polyvictimization in this population. Specifically, only one instance of experiencing any given category of PTE was counted, whereas individuals may have experienced multiple instances of each type of event (e.g., multiple instances of witnessing violence). With regards to reactions to these PTEs, approximately half of maternal report (53%) and just shy of three quarters of self-report (70%) indicated clinically elevated rates of PTSD symptomology. These rates are substantially higher than rates of PTSD symptomology following PTEs in the general population (i.e., 20% of youth exposed to trauma develop symptoms consistent with a diagnosis of PTSD; Alisic et al., 2014; McLaughlin et al., 2013; Saunders, 2003). Taken together, these findings highlight the elevated need for evidence-based interventions to address trauma-related symptomology within sheltered homeless youth.

Results of exploratory analyses highlighted substantial discrepancies between maternal and self-reported severity of PTSD symptoms and rates of exposure to most categories of PTEs. Self-report consistently indicated greater severity and rates of exposure types, except for witnessing violence within the family and emotional abuse which mothers rated as occurring more often than did youth. One possible explanation, for these findings is that parental report of PTEs that are witnessed by parents (those events which are most likely to occur in the home) are influenced by the parent’s own feelings of distress, either because they also experienced the trauma or because witnessing their child distressed is itself upsetting (Shemesh et al., 2003, 2005). In contrast, PTEs which occur outside the home are less likely to be witnessed by parents and therefore may only be reported if the youth informed their parent of the exposure. Additionally, it is possible that even when mothers are informed of the occurrence of PTEs they may minimize such exposures for fear of child welfare involvement. Alternatively, it is possible that these discrepancies are due to different parent and youth severity thresholds of what constitutes an exposure to PTEs. For example, a fender-bender might constitute a serious accident to a youth, but a parent may not consider an accident “serious” unless the youth went to the hospital. Regardless of the reason, the substantial discrepancies between maternal and self-reported PTSD symptomology highlight the importance of thorough multi-informant ratings of youth symptomology. These findings are in line with previous evidence that self-report of internalizing symptoms offers unique information beyond that obtained from parental report (e.g., Hope et al., 1999).

### Treatment Completion and Satisfaction

The access to and transportability of evidence-based interventions to those individuals with the greatest needs are significant issues in the intervention field (Herschell et al., 2004; Silverman & Kurtines, 2004). Families experiencing homelessness are arguably the population with the greatest physical, medical, and mental health needs (Arangua et al., 2005; Bassuk & Friedman, 2005; Lee et al., 2010). Specifically, 12.5% of youth experiencing homelessness have high levels (≥ 4) of ACES (Felitti et al., 1998), 20% have clinically significant emotional problems (Bassuk & Friedman, 2005), and 18% meet criteria for PTSD (Stewart et al., 2004). Despite this, in the past 20 years there has been a gap in the literature as to how best to address the mental health needs of sheltered youth and their families (Herschell et al., 2004; Silverman & Kurtines, 2004). This study represents a critical step towards identifying and addressing the elevated needs of youth experiencing homelessness by examining the feasibility, acceptability, and efficacy of administering both assessments and TF-CBT treatment within a shelter environment.

Completion rates of TF-CBT in the present study (72%), delivered within a homeless shelter, were comparable to or slightly better than those of previous trials of TF-CBT which have typically documented completion rates ranging from 55 to 75% (Cohen et al., 2005; Eslinger et al., 2014; Yasinski et al., 2016). Furthermore, maternal report indicated high rates of satisfaction with treatment, with the majority of families reporting that they would recommend the treatment to others. Thus, an appropriately resourced shelter which is trained and staffed to provide evidence-based services in-house has the substantial advantage of bypassing common barriers to providing interventions to this population, including familial transportation to and from services, familial engagement in the face of multiple complex life stressors, and parental time limitations. Providing free in-house services allowed for flexibility with regard to scheduling and rescheduling sessions, greater insight into the needs of the families, and a greater ability to build rapport as shelter staff were engaged with families on a daily basis outside of sessions; each of which helped address the well documented attendance difficulties of families participating in clinical services (e.g., Axford et al., 2012; Baker et al., 2011; Nock & Ferriter, 2005). Finally, the use of time-limited intervention also likely contributed to the reduced dropout rates. Specifically, the time-limited format addressed difficulties associated with sustaining proximity to therapeutic services for families in transition (Culhane et al., 2007) and associated with the burden of extensive time commitments from parents who were already overburdened. Taken together, the feasibility of training shelter staff to administer assessments and treatment with fidelity and obtaining relatively high completion rates given the at-risk nature of this population highlight the transportability of TF-CBT to a shelter setting for this vulnerable population of youth and families.

### Treatment Efficacy

Consistent with the original hypothesis, TF-CBT resulted in a reduced severity of PTSD symptomology based on both maternal and self-report as well as a reduction in the number of youth who fell within the clinically elevated range for PTSD. Further investigation revealed that these reductions were largely attributable to reduced symptoms of reexperiencing and arousal. Overall, these results offer promising evidence that even amongst this most at-risk population, TF-CBT can result in substantial improvements in trauma-related symptomology. These findings are particularly striking given that youth experiencing homelessness/poverty are at a substantial elevated risk for exposure to ACES (Halfon et al., 2017) in addition to PTEs. TF-CBT was effective at reducing trauma-related symptoms secondary to not only exposure to PTEs, but also secondary to elevated rates of ACEs/life stressors.

With regard to the symptoms of reexperiencing, research indicates that these symptoms typically follow the presence or recall of stimuli after a trauma, which during the trauma signaled the onset of trauma or a “turn for the worse” (Ehlers et al., 2004). These stimuli trigger reexperiencing if the individual has not put the trauma memories within temporal context (e.g., “this stimuli/memory occurred within the context of my broader trauma narrative and is not a warning signal for danger presently”; e.g., Ehlers & Clark, 2000). Given the attention paid to the trauma narrative within TF-CBT, which allows the individual to process the events that occurred in detail and within a temporal context, it follows that substantial reductions would be seen in reexperiencing symptoms. With regards to symptoms of arousal, these symptoms are the most external of the PTSD symptoms (e.g., angry outbursts, reckless behavior, sleep disruption) and therefore the easiest for parents to monitor without insight from their child. As such, it is possible that mothers were more likely to notice reductions in arousal symptoms in their child than symptoms which are more internalizing or emotion and thought related.

Consistent with our original hypothesis, TF-CBT also resulted in improvements in externalizing behaviors in the intent to treat group. However, follow-up analyses indicated that this effect is primarily due to changes in externalizing behaviors amongst elementary school-aged children, but not older adolescents. These findings are likely attributable to differences in the presentation of trauma-related symptoms across development (e.g., Yule & Smith, 2015). In fact, at pre-intervention elementary school-age children had a substantially higher mean score on the ECBI than did junior high and high school-age adolescents (*M* = 107.50, *SD* = 49.52 and *M* = 83.84, *SD* =  85.20, respectively), suggesting that older youth are less likely to express trauma-related reactions through externalizing behaviors than are younger youth, and therefore may require less focus on such symptoms in treatment. This finding is consistent with the DSM 5 accommodations which allow for different presentations of symptoms of PTSD in young children (American Psychiatric Association, 2013). However, the DSM 5 makes such accommodations only for children under the age of 6, and findings from this study suggest that throughout elementary school clinical presentation of trauma-reactions may continue to differ substantively from those of adolescents (and adults). As such, future research would benefit from continued investigation of the developmental trajectory of trauma-related symptomology.

Contrary to our original hypothesis, greater exposure to PTE types was associated with greater treatment response, such that youth who were exposed to less than four types of PTEs consistently benefited less from TF-CBT than did youth with exposure to 4 or more types of PTEs. It is important to note that those youth with four or more exposure types also had substantially higher pre-intervention ratings of trauma symptom severity, meaning that this group had a greater room for improvements to be seen. However, given that post-intervention severity scores across all three groups (0, 1–3, and 4 or more PTE exposure types) fell at subclinical levels for both maternal and self-report, it is clear that TF-CBT offers a viable and efficacious treatment option even amongst those youth with the most extensive exposures to and severe symptomology following PTE. In fact, these results suggest that TF-CBT is likely to be the most effective for treating trauma amongst those youth with the most extensive exposures to PTEs.

Notably, these findings are somewhat inconsistent with the broader literature, which has demonstrated that greater exposure to ACES (≥ 4) results in poorer response to treatment for internalizing symptomology (e.g., Hayden & Klein, 2001; Nanni et al., 2012) and that individuals with more complex trauma histories often require more extensive courses of TF-CBT (i.e., 16–25 sessions; Cohen & Mannarino, 2016). One possible explanation for these discrepancies with the broader literature is that whereas previous studies have focused on the incidence of exposures to PTE (i.e., the number of times a youth experienced physical abuse), the present study focused on exposure to different types/categories of trauma. As such, future work would benefit from examining whether TF-CBT continues to be equivalently effective at addressing trauma symptoms amongst youth experiencing homelessness with the greatest incidence of exposures to PTEs.

### Limitations

In terms of limitations, first, it is important to note that the study did not include a waitlist control. Given the high clinical needs of this population, it was deemed unethical to withhold treatment for a waitlist control. In particular, given the transient nature of the homeless population, there were concerns that asking families to wait for treatment would have been prohibitive, such that many families would have relocated before services became available to them. Now that the efficacy and feasibility of implementing TF-CBT within the context of a shelter has been established, future research would benefit from comparing TF-CBT to alternative treatment options via a randomized control trial, within the homeless shelter context. This will enable an examination of whether there are other interventions which work as effectively as TF-CBT. Second, this study did not include a homed comparison group. Future studies would benefit from a direct comparison of trauma and response to trauma-informed intervention between a sample of youth experiencing homelessness and their homed peers. Finally, it is important to acknowledge that findings from the present study cannot speak to the long-term maintenance of TF-CBT improvements in outcomes, as no follow-up data was collected in the present study. This was due to families exiting the shelter as well as limitations with regard to shelter resources.

### Clinical Implications

In terms of clinical implications, the current study demonstrates the importance of offering evidence-based assessments to detect and address the clinical needs of youth experiencing homelessness. Further, it demonstrates the feasibility, acceptability, and effectiveness of embedding evidence-based treatment programs within the context of a homeless shelter and other shelter environments, such as domestic violence shelters and transitional housing. The findings indicate that sheltered youth and their families can see substantial benefits from time-limited TF-CBT. While it is important to acknowledge that most homeless shelters face high staff turnover, limited resources, and minimal access to evidence-based programs (Gewirtz & August, 2008), the results from the present study are promising in suggesting that evidence-based programs to address the mental health needs and trauma of youth can feasibly be implemented if shelters are appropriately resourced, staffed, and trained. There is growing awareness of the elevated mental health needs of youth experiencing homelessness (Committee on Community Health Services, 1996; Weinreb et al., 1998) as well as the lack of access to quality mental health interventions (Bassuk & Friedman, 2005). The results of the present study demonstrate the potential for building not only community-university partnerships, but also other community-based provider partnerships, to develop evidence-based programs and better meet the needs of this most at-risk and underserved population. The implications for other community providers serving at-risk, marginalized youth and families to address the trauma of racial, ethnic, gender, and social and economic inequities and improve community health are broader still.

## Footnotes

1 Estimates of homelessness vary across reporting agencies, based on how restrictive their definition of homelessness are. For instance, the Department of Education has a broad definition of homelessness (e.g., including youth who are sharing the housing of other persons, living in motels, or camping grounds, and/or living in emergency or transitional shelters). More restrictive definitions of homelessness, such as that made by the Department of Housing and Urban Development (HUD), report far fewer youth experiencing homelessness (i.e., 580,466 people experienced homelessness in the United States, of whom 171,575 were families and youth; U.S. Department of HUD, 2019).

2 This data was collected for the 2017–2018 school year. This number *does not* reflect the totality of youth experiencing homelessness, as it only includes students enrolled in public school districts or local educational agencies.

3 Based upon Fletcher’s (1996) meta-analysis, PTSD was diagnosed in 39% of preschoolers (< 7 years old), 33% of school-aged children (6–12 years), and 27% of teenagers (> 12 years old).

4 Of note, few studies examined in the Alisic et al. (2014) meta-analysis (*k* = 4) examined parent-report of PTSD symptoms in isolation of youth self-report. Variation in effects across studies led to large *SD*, which resulted in non-significant differences between youth- and parent-based effects despite a large discrepancy between effect sizes.

5 Thirty-seven youth (11.6%) reportedly did not experience a potentially traumatic event prior to entry to the homeless shelter.

6 To address trauma-related symptoms in children below the age of 5, these children and their families were offered alternative intervention services which were more developmentally appropriate/validated for use in early childhood (e.g., Parent–Child Interaction Therapy; Eyberg et al., 2001). See (Graziano et al., 2020) for a description of services provided to children under the age of five.

7 Correlations between sex, maternal education, child race and ethnicity with maternal report of trauma severity (0.02, 0.08, 0.01, − 0.07, respectively), self-report of trauma severity (0.04, 0.07, − 0.03, .08 respectively), ECBI raw score (0.09, 0.07, − 0.03, − 0.02, respectively), and parenting stress total score (0.02, 0.02, − 0.01, 0.00, respectively) at post-intervention were all not significant (*p* > 0.05).

## References

[CR1] Alisic E, Zalta AK, Van Wesel F, Larsen SE, Hafstad GS, Hassanpour K, Smid GE (2014). Rates of post-traumatic stress disorder in trauma-exposed children and adolescents: Meta-analysis. The British Journal of Psychiatry.

[CR2] American Psychiatric Association (2013). Diagnostic and statistical manual of mental disorders.

[CR3] Arangua L, Andersen R, Gelberg L (2005). The health circumstances of homeless women in the United States. International Journal of Mental Health.

[CR4] Auslander W, McGinnis H, Tlapek S (2017). Adaptation and implementation of a trauma-focused cognitive behavioral intervention for girls in child welfare. American Journal of Orthopsychiatry.

[CR5] Axford N, Lehtonen M, Kaoukji D, Tobin K, Berry V (2012). Engaging parents in parenting programs: Lessons from research and practice. Children and Youth Services Review.

[CR6] Baker CN, Arnold DH, Meagher S (2011). Enrollment and attendance in a parent training prevention program for conduct problems. Prevention Science.

[CR7] Bassuk, E. L., & Friedman, S. M. (2005). Facts on trauma and homeless children. In *National child traumatic stress network*. Retrieved from https://www.nctsn.org/sites/default/files/resources//facts_on_trauma_and_homeless_children.

[CR8] Bassuk EL, DeCandia CJ, Beach CA, Berman F (2014). America's youngest outcasts: A report card on child homelessness.

[CR9] Bassuk EL, Richard MK, Tsertsvadze A (2015). The prevalence of mental illness in homeless children: A systematic review and meta-analysis. Journal of the American Academy of Child & Adolescent Psychiatry.

[CR10] Becker SP, Kerig PK (2011). Posttraumatic stress symptoms are associated with the frequency and severity of delinquency among detained boys. Journal of Clinical Child & Adolescent Psychology.

[CR11] Berger R, Gelkopf M (2009). School-based intervention for the treatment of tsunami-related distress in children: A quasi-randomized controlled trial. Psychotherapy and Psychosomatics.

[CR12] Bolton D, O’Ryan D, Udwin O, Boyle S, Yule W (2000). The long-term psychological effects of a disaster experienced in adolescence: II: General psychopathology. Journal of Child Psychology and Psychiatry.

[CR125] Brestan EV, Jacobs JR, Rayfield AD, Eyberg SM (1999). A consumer satisfaction measure for parent-child treatments and its relation to measures of child behavior change. Behavior therapy.

[CR13] Brown E, Cohen JA, Mannarino AP (2020). Trauma-focused cognitive behavioral therapy: The role of caregivers. Journal of Affective Disorders.

[CR14] Bui E, Ohye B, Palitz S, Olliac B, Goutaudier N, Raynaud JP, Kounou KB, Stoddard FJ (2014). Acute and chronic reactions to trauma in children and adolescents. IACAPAP e-Textbook of Child and Adolescent Mental Health.

[CR15] Carrion VG, Weems CF, Ray R, Reiss AL (2002). Toward an empirical definition of pediatric PTSD: The phenomenology of PTSD symptoms in youth. Journal of the American Academy of Child & Adolescent Psychiatry.

[CR16] Cary CE, McMillen JC (2012). The data behind the dissemination: A systematic review of trauma-focused cognitive behavioral therapy for use with children and youth. Children and Youth Services Review.

[CR17] Casey EC, Shlafer RJ, Masten AS (2015). Parental incarceration as a risk factor for children in homeless families. Family Relations.

[CR18] Caspi A, McClay J, Moffit TE, Mill J, Martin J, Craig W, Taylor A, Poulton R (2002). Role of genotype in the cycle of violence in maltreated children. Science.

[CR19] Caspi A, Moffitt TE, Morgan J, Rutter M, Taylor A, Arseneault L, Tully L, Jacobs C, Kim-Cohen J, Polo-Tomas M (2004). Maternal expressed emotion predicts children’s antisocial behavior problems: Using MZ-twin differences to identify environmental effects on behavioral development. Developmental Psychology.

[CR20] Ceballo R, Dahl T, Aretakis M, Ramirez C (2001). Inner-city children’s exposure to community violence: How much do parents know?. Journal of Marriage and Family.

[CR21] Cohen, J.A. (2005). Treating PTSD in children exposed to domestic violence [NCT00183326]. Retrieved May 15, 2018, from https://www.clinicaltrials.gov/ct2/show/NCT00183326.

[CR22] Cohen JA, Mannarino AP (1996). A treatment outcome study for sexually abused preschooler children: Initial findings. Journal of the American Academy of Child and Adolescent Psychiatry.

[CR23] Cohen JA, Mannarino AP (2008). Trauma-focused cognitive behavioural therapy for children and parents. Child and Adolescent Mental Health.

[CR24] Cohen JA, Deblinger E, Mannarino AP, Steer R (2004). A multi-site randomized controlled trial for sexually abused children with PTSD symptoms. Journal of the American Academy of Child and Adolescent Psychiatry.

[CR25] Cohen JA, Mannarino AP, Knudsen K (2004). Treating childhood traumatic grief: A pilot study. Journal of the American Academy of Child & Adolescent Psychiatry.

[CR26] Cohen JA, Mannarino AP, Knudsen K (2005). Treating sexually abused children: One year follow-up of a randomized controlled trial. Child Abuse and Neglect.

[CR27] Cohen JA, Mannarino AP, Staron V (2006). Modified cognitive behavioral therapy for childhood traumatic grief (CBT-CTG): A pilot study. Journal of the American Academy of Child and Adolescent Psychiatry.

[CR28] Cohen JA, Mannarino AP, Deblinger E (2010). Trauma-focused cognitive-behavioral therapy for traumatized children. Evidence-Based Psychotherapies for Children and Adolescents.

[CR29] Cohen JA, Mannarino AP, Iyengar S (2011). Community treatment of posttraumatic stress disorder for children exposed to intimate partner violence: A randomized controlled trial. Archives of Pediatrics and Adolescent Medicine.

[CR30] Cohen JA, Mannarino AP, Jankowski K, Rosenberg S, Kodya S, Wolford GL (2016). A randomized implementation study of trauma-focused cognitive behavioral therapy for adjudicated teens in residential treatment facilities. Child Maltreatment.

[CR31] Cohen JA, Mannarino A, Deblinger E (2016). Treating trauma and traumatic grief in children and adolescents.

[CR32] Cowal K, Shinn M, Weitzman BC, Stojanovic D, Labay L (2002). Mother–child separations among homeless and housed families receiving public assistance in New York City. American Journal of Community Psychology.

[CR33] Cronholm PF, Forke CM, Wade R, Bair-Merritt MH, Davis M, Harkins-Schwarz M, Pachter LM, Fein JA (2015). Adverse childhood experiences: Expanding the concept of adversity. American Journal of Preventive Medicine.

[CR34] Cuffe SP, Addy CL, Garrison CZ, Waller JL, Jackson KL, McKeown RE, Chilappagari S (1998). Prevalence of PTSD in a community sample of older adolescents. Journal of the American Academy of Child & Adolescent Psychiatry.

[CR35] Culhane DP, Metraux S, Park JM, Schretzman M, Valente J (2007). Testing a typology of family homelessness based on patterns of public shelter utilization in four US jurisdictions: Implications for policy and program planning. Housing Policy Debate.

[CR36] Deblinger E, Steer R, Lippmann J (1999). Maternal factors associated with sexually abused children's psychosocial adjustment. Child Maltreatment.

[CR37] Department of Housing and Urban Development. (2019). CoC homeless populations and subpopulations reports. In* HUD exchange*. Retrieved from www.hudexchange.info/programs/coc/coc-homeless-populations-and-subpopulations-reports/.

[CR38] Ertl V, Pfeiffer A, Schauer E, Elber T, Neuner F (2011). Community-implemented trauma therapy for former child soldiers in Northern Uganda: A randomized controlled trial. JAMA.

[CR39] Ehlers A, Clark DM (2000). A cognitive model of posttraumatic stress disorder. Behaviour Research and Therapy.

[CR40] Ehlers A, Hackmann A, Michael T (2004). Intrusive re-experiencing in post-traumatic stress disorder: Phenomenology, theory, and therapy. Memory.

[CR41] Eisenberg N, Fabes RA, Murphy BC (1996). Parents' reactions to children's negative emotions: Relations to children's social competence and comforting behavior. Child Development.

[CR42] Eslinger JG, Sprang G, Otis MD (2014). Child and caregiver dropout in child psychotherapy for trauma. Journal of Loss and Trauma.

[CR43] Eyberg SM, Funderburk BW, Hembree-Kigin TL, McNeil CB, Querido JG, Hood KK (2001). Parent-child interaction therapy with behavior problem children: One and two year maintenance of treatment effects in the family. Child & Family Behavior Therapy.

[CR126] Eyberg SM, Ross AW (1978). Assessment of child behavior problems: The validation of a new inventory. Journal of Clinical Child & Adolescent Psychology.

[CR44] Felitti VJ, Anda RF, Nordenberg D, Williamson DF, Spitz AM, Edwards V, Marks JS (1998). Relationship of childhood abuse and household dysfunction to many of the leading causes of death in adults: The Adverse Childhood Experiences (ACE) Study. American Journal of Preventive Medicine.

[CR45] Fletcher KE, Mash EJ, Barkley R (1996). Childhood posttraumatic stress disorder. Child psychopathology.

[CR46] Finkelhor D, Ormrod RK, Turner HA (2007). Polyvictimization and trauma in a national longitudinal cohort. Development and Psychopathology.

[CR47] Finkelhor D, Ormrod RK, Turner HA (2009). Lifetime assessment of poly-victimization in a national sample of children and youth. Child Abuse & Neglect.

[CR48] Fitton L, Yu R, Fazel S (2020). Childhood maltreatment and violent outcomes: A systematic review and meta-analysis of prospective studies. Trauma, Violence, & Abuse.

[CR49] Fivush R (1998). Children's recollections of traumatic and nontraumatic events. Development and Psychopathology.

[CR50] Gewirtz AH, August GJ (2008). Incorporating multifaceted mental health prevention services in community sectors-of-care. Clinical Child and Family Psychology Review.

[CR51] Goodman LA, Saxe L, Harvey M (1991). Homelessness as psychological trauma: Broadening perspectives. American Psychologist.

[CR52] Grasso DJ, Saunders BE, Williams LM, Hanson R, Smith DW, Fitzgerald MM (2013). Patterns of multiple victimization among maltreated children in Navy families. Journal of Traumatic Stress.

[CR53] Graziano PA, Spiegel JA, Arcia E (2020). Early assessment and intervention for families experiencing homelessness: A randomized trial comparing two parenting programs. medRxiv.

[CR54] Green BL, Wilson JP, Lindy JD, Figley CR (1985). Conceptualizing posttraumatic stress disorder: A psycho-social framework. Trauma and its wake: The study and treatment of post-traumatic stress disorder.

[CR55] Gutermann J, Schreiber F, Matulis S, Schwartzkopff L, Deppe J, Steil R (2016). Psychological treatments for symptoms of posttraumatic stress disorder in children, adolescents, and young adults: A meta-analysis. Clinical Child and Family Psychology Review.

[CR56] Halfon N, Larson K, Son J, Lu M, Bethell C (2017). Income inequality and the differential effect of adverse childhood experiences in US children. Academic Pediatrics.

[CR57] Hayden EP, Klein DN (2001). Outcome of dysthymic disorder at 5-year follow-up: The effect of familial psychopathology, early adversity, personality, comorbidity, and chronic stress. American Journal of Psychiatry.

[CR58] Herschell AD, McNeil CB, McNeil DW (2004). Clinical child psychology's progress in disseminating empirically supported treatments. Clinical Psychology: Science and Practice.

[CR59] Hoagwood, K. E., Radigan, M., Rodriguez, J., Levitt, J. M., Fernandez, D., & Foster, J. (2006). *Final report on the Child and Adolescent Trauma Treatment Consortium (CATS) Project for the Substance Abuse and Mental Health Services Administration.* New York State Office of Mental Health, and Columbia University.

[CR60] Holm S (1979). A simple sequentially rejective multiple test procedure. Scandinavian Journal of Statistics.

[CR61] Hoogsteder LM, Ten Thije L, Schippers EE, Stams GJJ (2021). A meta-analysis of the effectiveness of EMDR and TF-CBT in reducing trauma symptoms and externalizing behavior problems in adolescents. International Journal of Offender Therapy and Comparative Criminology.

[CR62] Hope TL, Adams C, Reynolds L, Powers D, Perez RA, Kelley ML (1999). Parent vs. self-report: Contributions toward diagnosis of adolescent psychopathology. Journal of Psychopathology and Behavioral Assessment.

[CR63] Hunt TK, Slack KS, Berger LM (2017). Adverse childhood experiences and behavioral problems in middle childhood. Child Abuse & Neglect.

[CR64] Kaplan I, Stolk Y, Valibhoy M, Tucker A, Baker J (2016). Cognitive assessment of refugee children: Effects of trauma and new language acquisition. Transcultural Psychiatry.

[CR65] Keeshin BR, Campbell K (2011). Screening homeless youth for histories of abuse: Prevalence, enduring effects, and interest in treatment. Child Abuse & Neglect.

[CR66] King NJ, Tonge BJ, Mullen P, Myerson N, Heyne D, Rollings S, Martin R, Ollendick TH (2000). Treating sexually abused children with posttraumatic stress symptoms: A randomized clinical trial. Journal of the American Academy of Child and Adolescent Psychiatry.

[CR67] Kliethermes MD, Drewry K, Wamser-Nanney R, Landolt MA, Cloitre M, Schnyder U (2017). Trauma-focused cognitive behavioral therapy. Evidence-based treatments for trauma related disorders in children and adolescents.

[CR68] Kowalik J, Weller J, Venter J, Drachman D (2011). Cognitive behavioral therapy for the treatment of pediatric posttraumatic stress disorder: A review and meta-analysis. Journal of Behavior Therapy and Experimental Psychiatry.

[CR69] Lee S, August G, Gewirtz A, Klimes-Dougan B, Bloomquist M, Realmuto G (2010). Identifying unmet mental health needs in children of formerly homeless mothers living in a supportive housing community sector of care. Journal of Abnormal Child Psychology.

[CR70] Little, R., & Yau, L. (1996). Intent-to-treat analysis for longitudinal studies with drop-outs. *Biometrics,* 1324–1333. 8962456

[CR71] Maschi T, Baer J, Morrissey MB, Moreno C (2013). The aftermath of childhood trauma on late life mental and physical health: A review of the literature. Traumatology.

[CR73] Mash, E. J., & Terdal, L. G. (1997).* Assessment of child and family disturbance*. Assessment of childhood disorders.

[CR74] Masten AS (2011). Resilience in children threatened by extreme adversity: Frameworks for research, practice, and translational synergy. Development and Psychopathology.

[CR75] Masten AS, Miliotis D, Graham-Bermann SA, Ramirez ML, Neemann J (1993). Children in homeless families: Risks to mental health and development. Journal of Consulting and Clinical Psychology.

[CR76] Mavranezouli I, Megnin-Viggars O, Daly C, Dias S, Stockton S, Meiser-Stedman R, Trickey D, Pilling S (2020). Research review: Psychological and psychosocial treatments for children and young people with post-traumatic stress disorder: A network meta-analysis. Journal of Child Psychology and Psychiatry.

[CR77] McLaughlin KA, Koenen KC, Hill ED, Petukhova M, Sampson NA, Zaslavsky AM, Kessler RC (2013). Trauma exposure and posttraumatic stress disorder in a national sample of adolescents. Journal of the American Academy of Child & Adolescent Psychiatry.

[CR78] Meiser-Stedman R, Smith P, Glucksman E, Yule W, Dalgleish T (2008). The posttraumatic stress disorder diagnosis in preschool- and elementary school-age children exposed to motor vehicle accidents. American Journal of Psychiatry.

[CR79] Merrick MT, Ports KA, Ford DC, Afifi TO, Gershoff ET, Grogan-Kaylor A (2017). Unpacking the impact of adverse childhood experiences on adult mental health. Child Abuse & Neglect.

[CR80] Morgan L, Scourfield J, Williams D, Jasper A, Lewis G (2003). The Aberfan disaster: 33-year follow-up of survivors. British Journal of Psychiatry.

[CR81] Morina N, Koerssen R, Pollet TV (2016). Interventions for children and adolescents with posttraumatic stress disorder: A meta-analysis of comparative outcome studies. Clinical Psychology Review.

[CR82] Nanni V, Uher R, Danese A (2012). Childhood maltreatment predicts unfavorable course of illness and treatment outcome in depression: A meta-analysis. American Journal of Psychiatry.

[CR83] National Alliance to End Homelessness. (2019) New federal policy proposals will hurt survivors of domestic violence. Retrieved from https://endhomelessness.org/new-federal-policy-proposals-will-hurt-survivors-of-domestic-violence/.

[CR84] National Center on Family Homelessness. (1999). Homeless children: America’s new outcasts. retrieved from https://www.nn4youth.org/wp-content/uploads/A2HomelessChildren.pdf

[CR85] Nock MK, Ferriter C (2005). Parent management of attendance and adherence in child and adolescent therapy: A conceptual and empirical review. Clinical Child and Family Psychology Review.

[CR86] Nurcombe B (2000). Child sexual abuse I: Psychopathology. Australian & New Zealand Journal of Psychiatry.

[CR87] Obradović J, Bush NR, Stamperdahl J, Adler NE, Boyce WT (2010). Biological sensitivity to context: The interactive effects of stress reactivity and family adversity on socioemotional behavior and school readiness. Child Development.

[CR88] Oransky M, Hahn H, Stover CS (2013). Caregiver and youth agreement regarding youths’ trauma histories: Implications for youths’ functioning after exposure to trauma. Journal of Youth and Adolescence.

[CR89] Panter-Brick C (2004). Homelessness, poverty, and risks to health: Beyond at risk categorizations of street children. Children’s Geographies.

[CR90] Pityaratstian N, Piyasil V, Ketumarn P (2015). Randomized controlled trial of group cognitive behavioural therapy for post-traumatic stress disorder in children and adolescents exposed to tsunami in Thailand. Behavioural and Cognitive Psychotherapy.

[CR92] Pollio E, Deblinger E (2017). Trauma-focused cognitive behavioural therapy for young children: Clinical considerations. European Journal of Psychotraumatology.

[CR91] Rubin, D. B. (1988). An overview of multiple imputation. In *Proceedings of the survey research methods section of the American statistical association* (pp. 79–84). Princeton, NJ, USA: Citeseer.

[CR93] Ruf M, Schauer M, Neuner F, Catani C, Schauer E, Elbert T (2010). Narrative exposure therapy for 7-to 16-year-olds: A randomized controlled trial with traumatized refugee children. Journal of Traumatic Stress.

[CR94] Saunders BE (2003). Understanding children exposed to violence: Toward an integration of overlapping fields. Journal of Interpersonal Violence.

[CR95] Saunders BE, Adams ZW (2014). Epidemiology of traumatic experiences in childhood. Child and Adolescent Psychiatric Clinics.

[CR96] Shemesh E, Keshavarz R, Leichtling NK, Weinberg E, Mousavi A, Sadow K, Newcorn JH, Schmeidler J, Yehuda R (2003). Pediatric emergency department assessment of psychological trauma and posttraumatic stress. Psychiatric Services.

[CR97] Shemesh E, Newcorn JH, Rockmore L, Shneider BL, Emre S, Gelb BD, Rapaport R, Noone SA, Annunziato R, Schmeidler J, Yehuda R (2005). Comparison of parent and child reports of emotional trauma symptoms in pediatric outpatient settings. Pediatrics.

[CR98] Shelton KH, Taylor PJ, Bonner A, van den Bree M (2015). Risk factors for homelessness: Evidence from a population-based study. Psychiatric Services.

[CR99] Silverman WK, Kurtines WM (2004). Research progress on effectiveness, transportability, and dissemination of empirically supported treatments: Integrating theory and research. Clinical Psychology: Science and Practice.

[CR100] Silverman WK, Ortiz CD, Viswesvaran C, Burns BJ, Kolko DJ, Putnam FW, Amaya-Jackson L (2008). Evidence-based psychosocial treatments for children and adolescents exposed to traumatic events. Journal of Clinical Child & Adolescent Psychology.

[CR101] Smith P, Yule W, Perrin S, Tranah T, Dalgleish T, Clark DM (2007). Cognitive-behavioral therapy for PTSD in children and adolescents: A preliminary randomized controlled trial. Journal of the American Academy of Child & Adolescent Psychiatry.

[CR102] Stein JA, Leslie MB, Nyamathi A (2002). Relative contributions of parent substance use and childhood maltreatment to chronic homelessness, depression, and substance abuse problems among homeless women: Mediating roles of self-esteem and abuse in adulthood. Child Abuse & Neglect.

[CR103] Stein BD, Jaycox LH, Kataoka SH, Wong M, Tu W, Elliott MN, Fink A (2003). A mental health intervention for schoolchildren exposed to violence—a randomized controlled trial. JAMA.

[CR104] Stewart AJ, Steiman M, Cauce AM, Cochran BN, Whitbeck LB, Hoyt DR (2004). Victimization and posttraumatic stress disorder among homeless adolescents. Journal of the American Academy of Child & Adolescent Psychiatry.

[CR105] Stover CS, Hahn H, Im JJY, Berkowitz S (2010). Agreement of parent and child reports of trauma exposure and symptoms in the early aftermath of a traumatic event. Psychological Trauma: Theory, Research, Practice, and Policy.

[CR106] Toro PA, Trickett EJ, Wall DD, Salem DA (1991). Homelessness in the United States: An ecological perspective. American Psychologist.

[CR116] United Nations Economic and Social Council. (2019). Report of the Secretary General. Affordable Housing and Social Protection Systems for all to Address Homelessness. Retrieved from https://digitallibrary.un.org/record/3840349?ln=en

[CR117] Vilsack, T., Pritzker, P., Carter, A., Duncan, A., Moniz, E., Burwell, S.M., Johnson, J., Castro, J., Jewell, S., Lynch, L., Perez, T., Foxx, A, McDonald, R., Spencer, W., Roth, D.T., Donovan, S., Colvin, C., Brennan, M., Rogers, M, & Doherty, M. (2015) United States Interagency Council on Homelessness.

[CR107] Von Hippel PT (2020). How many imputations do you need? A two-stage calculation using a quadratic rule. Sociological Methods & Research.

[CR108] Walker JL, Carey PD, Mohr N, Stein DJ, Seedat S (2004). Gender differences in the prevalence of childhood sexual abuse and in the development of pediatric PTSD. Archives of Women’s Mental Health.

[CR109] Weinreb L, Goldberg R, Bassuk E, Perloff J (1998). Determinants of health and service use patterns in homeless and low-income housed children. Pediatrics.

[CR110] Wenocur K, Parkinson-Sidorski M, Snyder S (2016). Provision of child trauma services in emergency family housing (practice note). Families in Society.

[CR111] Yasinski C, Hayes AM, Ready CB, Cummings JA, Berman IS, McCauley T, Webb C, Deblinger E (2016). In-session caregiver behavior predicts symptom change in youth receiving trauma-focused cognitive behavioral therapy (TF-CBT). Journal of Consulting and Clinical Psychology.

[CR112] Yule W, Smith P (2015). Post traumatic stress disorder. Rutter's child and adolescent psychiatry.

[CR113] Yule W, Bolton D, Udwin O, Boyle S, O’Ryan D, Nurrish J (2000). The long-term psychological effects of a disaster experienced in adolescence: I: The incidence and course of PTSD. Journal of Child Psychology and Psychiatry.

[CR114] Zlotnick C (2009). What research tells us about the intersecting streams of homelessness and foster care. American Journal of Orthopsychiatry.

[CR115] Zlotnick C, Kronstadt D, Klee L (1998). Foster care children and family homelessness. American Journal of Public Health.

[CR118] Committee on Community Health Services (1996). Health needs of homeless children and families. Pediatrics.

[CR119] Shannon, M. P., Lonigan, C. J., Finch, Jr, A. J., & Taylor, C. M. (1994). Children exposed to disaster: I. Epidemiology of post-traumatic symptoms and symptom profiles.* Journal of the American Academy of Child and Adolescent Psychiatry*, *33*(1), 80-93. 10.1097/00004583-199401000-00012 8138525

[CR120] Kirkpatrick H, Byrne C (2009). A narrative inquiry: Moving on from homelessness for individuals with a major mental illness. Journal of Psychiatric and Mental Health Nursing.

[CR122] Masten AS, Sesma A, Si-Asar R, Lawrence C, Miliotis D, Dionne JA (1997). Educational risks for children experiencing homelessness. Journal of School Psychology.

[CR123] Wildeman C (2014). Parental incarceration, child homelessness, and the invisible consequences of mass imprisonment. The ANNALS of the American Academy of Political and Social Science.

